# Exploring the Retail Food Environment Surrounding Two Secondary Schools with Predominantly Pacific Populations in Tonga and New Zealand to Enable the Development of Mapping Methods Appropriate for Testing in a Classroom

**DOI:** 10.3390/ijerph192315941

**Published:** 2022-11-29

**Authors:** Alvina F. Pauuvale, Mark H. Vickers, Soana Pamaka, Dorothy Apelu, ‘Anaseini Fehoko, Malakai ‘Ofanoa, Jacquie L. Bay

**Affiliations:** 1 Liggins Institute, University of Auckland, Auckland 1142, New Zealand; 2 School of Population Health, University of Auckland, Auckland 1142, New Zealand; 3 Tamaki College, Auckland 1072, New Zealand; 4 Tonga High School, Nuku’alofa P.O. Box 53, Tonga

**Keywords:** noncommunicable diseases 1, retail food environment 2, Pacific 3, New Zealand 4, Tonga 5, school-based 6, Google tools 7, spatial mapping 8

## Abstract

Rates of noncommunicable diseases (NCDs) are disproportionately high among people of Pacific ethnicity. Nutrition-related environmental exposures including food access and quality contribute to the matrix of factors impacting risk. Preventative interventions in adolescence and the opportunity to integrate health promotion into school-based learning are often overlooked. This study tested the potential of a low-cost method to map the retail food environment in a 1 km radius of two secondary schools in low socioeconomic communities with predominantly Pacific populations, in Tonga and New Zealand (NZ). Mapping utilized Google Earth, Google Maps, government maps, and observations. A rubric was developed to categorize food quality. Outlets within a 1 km radius of each school, (Tonga, n = 150; NZ, n = 52) stocked predominantly unhealthy foods. The NZ data compared favorably to previous studies, indicating the method was valid. The Tongan data is novel and indicates that alternative strategies can be used when access to GIS-type tools is limited. The method produced visual data that has the potential to be analyzed using strategies appropriate for secondary schools. The method should now be tested in classrooms to assess its potential to support school-age students to engage in mapping and critiquing the retail food environment.

## 1. Introduction

The incidence of overweight, obesity and related noncommunicable diseases (NCDs), such as type 2 diabetes and heart disease, is disproportionately high amongst Pacific peoples in Pacific Island nations, New Zealand (NZ), Australia and the US [1]. Further, Pacific populations are impacted by high rates of overweight and obesity in childhood and adolescence, and in some cases, adolescent onset of type 2 diabetes [2,3,4,5]. 

In NZ, Pacific peoples represented 8.1% of the population in 2018, up from 7.4% in 2013. This population is young (median age 23.4 years) and urban dwelling; 64% live in the largest city, Auckland [6]. Pacific peoples have the lowest recorded median income and highest rates of adult (66%) and child (2–14 years; 30%) obesity compared to other ethnic groups in NZ [7]. 

Tonga is an upper-middle-income Pacific Island nation. The 2016 census recorded a population of 100,651, of which 74% live on the main island of Tongatapu; 97% are of Tongan ethnicity, and 23% live in the urban area of Nuku’alofa on Tongatapu. The median age of the population is 21.3 years [8]. In the adult population aged 25–64 years, 57% live with three to five NCD risk factors, 68% live with obesity, and a further 31% are overweight [9]. In the adolescent population aged 13–17 years, 56% are overweight and 25% obese [10]. 

A complex system of interactions between social, environmental, lifestyle, physiological and genetic factors contribute to the development of overweight, obesity and related NCDs [11]. Amongst this matrix, suboptimal diets have been identified as an important preventable risk factor [12]. Pacific peoples living in both developed and developing economies in the Pacific region are disproportionately impacted by economic poverty, which is associated with suboptimal diets [13]. Numerous dietary intake studies have demonstrated connections between changes in nutritional choices and rising levels of obesity in the Pacific region [1,14].

The retail food environment has a key role in influencing food choices. Neighborhood food environments such as ‘food deserts’ and ‘food swamps’ are positively associated with obesity status [15,16,17]. The retail food environment has two components. The community food environment measures the type, availability and accessibility of food outlets, while the consumer food environment measures the availability, prices, promotions and nutritional quality of produces available within stores [18]. Food deserts are areas with a low density of outlets supplying nutritious foods, whereas food swamps are characterized by a high density of outlets supplying unhealthy, high-calorie foods [19]. The retail food environment can be assessed using absolute or relative measures. Absolute measures indicate the density of particular types of retail outlets (e.g., supermarkets or fast food) within a defined area. Measures of relative healthy food access (RHFA) evaluate the percentage of healthy food outlets within an area’s overall retail food environment and are considered more insightful than absolute measures [20,21]. 

Mapping of the retail food environment in NZ has demonstrated significant associations between economically deprived communities and a high density of unhealthy food outlets [22]. To our knowledge, there is no published evidence of the mapping of the retail food environment in Tonga. 

Dietary habits that emerge during adolescence continue into adulthood and can have intergenerational impacts on health and wellbeing [23,24]. These habits contribute to the development of overweight and obesity. Once obesity is established, it is challenging to reverse the biological factors that support the ongoing condition in an individual [25]. Therefore, the adolescent life phase is identified as a highly influential point for NCD risk reduction strategies. We propose that the role of the retail food environment in influencing nutritional behaviors during adolescence may provide a context for school-based health promotion. 

Schools are a key site for interventions designed to develop health literacy and promote lifestyle behaviors that will impact the current and future health of adolescents and their future offspring [26]. To be sustainable such interventions must be informed by current pedagogy and practice and designed in partnership with schools to enable flexible integration into learning within core curriculum subjects and policy [27]. Appropriate pedagogical approaches can support the development of capabilities that integrate scientific, health and sociological literacies, which can be used to challenge adolescents to explore evidence of complex social issues such as NCD risk and identify actions that are relevant to their setting [28,29]. School-based activities that facilitate adolescents to explore the retail food environment surrounding their school and or home could stimulate learning that integrates the development of capabilities required for health literacy with learning objectives in core curriculum subjects such as health, science, and social sciences. Furthermore, when adolescents are involved in exploring local evidence relevant to social issues, they are more likely to act on their learning [30]. 

Evidence of associations between the retail food environment surrounding schools and obesity or nutritional behaviors in school-age children is variable. Consensus opinion indicates a requirement for further longitudinal investigation [31,32,33,34,35,36,37]. In NZ, mapping of the retail food environment surrounding schools has demonstrated greater accessibility of fast food, takeaway and convenience outlets surrounding schools in communities with high levels of economic poverty [38]. Adolescents (age 12–17 years) of Pacific ethnicity and adolescents of all ethnicities from families living with high burdens of economic poverty in NZ are more likely to purchase school lunch items and have money to spend on before and after school food purchases than other adolescents [39]. Furthermore, there is evidence of a positive association between food purchasing behaviors and body weight in NZ children [40]. Given the high levels of NCD risk and burden carried by Pacific populations, it is important that this evidence can be shared and discussed with teachers, parents, adolescents, and key stakeholders. We are not aware of any similar evidence regarding mapping of the retail food environment for schools in Tonga, where it is known that it is common for 13- to 17-year-old school students to purchase lunch (69%) and breakfast (66%) from the school canteen [10]. 

This study was designed to contribute to the process of constructing a school-based health promotion strategy that centers on the contribution of the retail food environment to the development of nutritional behaviors. We report on the development of a mapping tool that would be accessible in schools in two settings—one community in a developed economy with access to detailed internet mapping, and one community in a middle-income economy with access to less detailed internet mapping. We propose that prior to designing a program that integrates examination of the impact of the retail food environment on nutrition within core learning programs, teachers need access to:

1. opportunities to examine the potential to integrate health promotion into learning that promotes core curriculum objectives and can therefore be sustained within the school environment
2. opportunities to learn about the role of nutrition during adolescence on lifelong health and wellbeing, and the impact of the retail food environment on adolescent nutritional behaviors
3. evidence about the retail food environment in general, and specific evidence about the retail food environment surrounding their school community
4. a low-cost, easily accessible method of assessing the retail food environment that could be reliably used by adolescents in a school setting

To address points 3 and 4, this study aimed to identify whether a point in time snapshot of the retail food environment in a predetermined area surrounding a school could be obtained using minimal technologies, accessible by teachers in a school setting, and provide adequate and visualizable data for investigation by school-age students. By using two schools, we aimed to assess the accessibility of the method in geographically diverse settings with significant differences in technology access. The setting was two schools that serve predominantly low-income Pacific communities with very high rates of child, adolescent and adult overweight and obesity. 

Setting A, (NZ) is centered on a coeducational state (government) secondary school catering for 600 students aged 13–18 years in a residential suburb of East Auckland, approximately 1 km from the urban town center. The school population is predominantly (68%) of Pacific ethnicity, composed of Tongan 35%, Samoan 16%, Cook Islands 10%, Niuean 6%, Pacific other 1%. New Zealand schools are allocated a socioeconomic status (SES) decile based on the SES of families with children likely to populate that school [41]. The school is rated Decile 1 on a 1-10 scale where 1 represents the lowest SES category. 

Setting B (Tonga) is centered on a coeducational secondary school catering for 1200 students aged 11–18 years, 98% of whom are of Tongan ethnicity. It is situated on the main thoroughfare in the center of Nuku’alofa, the most densely populated and only urban area of Tonga. Nuku’alofa is located on the main island of Tongatapu and has a population of 24,000 (24% of the population of the island of Tongatapu) [8]. This school is one of three state-owned secondary schools in Tongatapu. Despite being a government school, students must apply to attend the school, which is recognized for academic excellence. While students who attend this school travel from all parts of Tongatapu, the majority are from Nuku’alofa. 

A range of spatial mapping methods has been previously reported in studies examining associations between the retail-food environment and proximity to schools [31,38,42]. We utilized evidence from these studies to design a methodological approach suitable for use in NZ and Tonga, thus enabling comparison. Resource differences between the settings were considered. Methods were developed with the potential for use by school students in settings with limited technology access. 

## 2. Materials and Methods

The data presented in this paper were collected over the period 2017 to 2018. 

### 2.1. Data Preparation 

**Figure 1:**
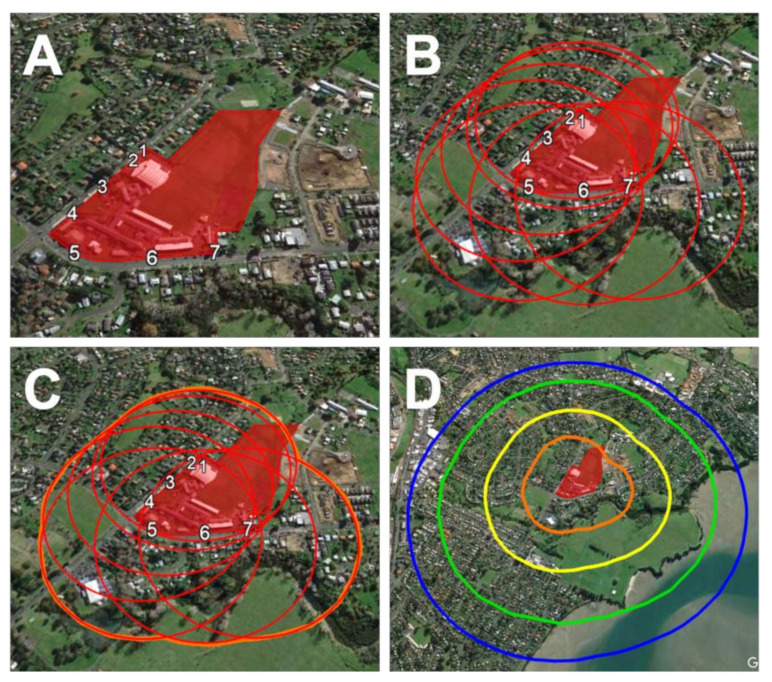
Preparation of the mapping area via Google Earth Pro, 2017; (**A**): school site and entries; (**B**,**C**): marking concentric intervals (250 m 500 m, 800 m, 1000 m) from entry points; (**D**)—final radial zones used for mapping.

The school sites and their surrounding area were mapped using Google Earth Pro supported by Google Maps [43]. The available maps on Google Earth were taken on 06/01/16 (Auckland, NZ) and 06/02/16 (Nuku’alofa, Tonga). The site of the school premises was identified, and the points of entry (pedestrian and vehicular) were marked (Figure 1A). From each entry point, concentric intervals of 250 m, 500 m, 800 m, and 1000 m were marked and connected to create circular boundaries (Figure 1B,C). These boundaries define the radial zones within each study site, used for virtual and physical observational mapping (Figure 1D). 

### 2.2. Data Collection

Food outlets within each map area were plotted. In NZ, this involved mapping via Google street-view, followed by confirmation via in-person observations. Google street-view was not available in Tonga at the time of data collection. A government map showing the positions of many food outlets was used in conjunction with in-person observations [44].

Food ‘outlets’ were defined as any shop selling foodstuff including grocery store, delicatessen, convenience store or market, provider of take-out foods, café, supermarket, bakery or restaurant. A rubric was created to support observation of the types of food, and relative proportion of food categories in each outlet (Table 1), categorizing food as: health-promoting (fruit, vegetables and whole-grain), risk-promoting (high-fat, high-sugar) and sugar-sweetened beverages (SSBs). This needed to be suitable for future use by adolescents, so could not be overly detailed. The decision to separate SSBs from the general category of risk promoting foods was based on the visibility of these foods to adolescents and similar separation in the Global School-based student Health Survey (GSHS) [45], which is used in Tonga, and the New Zealand Health Survey [46]. Observation was used to estimate the proportion of the total food in the store within each category.

### 2.3. Data Analysis

Data were analyzed using Microsoft Excel and IBM SPSS v28. 

A healthfulness rating was calculated for each food outlet using the observed ‘quality of food’ data and a simple scoring system that would be appropriate for use by school-age adolescents (Table 2). 

For example, food outlet X:

- 10% health-promoting foods|Rating = 1
- 55% risk promoting foods|Rating = 3
- 35% sugar-sweetened beverages|Rating = 2
- Total healthfulness rating score = 6

Scores were categorized using predetermined ranges (Table 3). Food outlets were marked on the maps using colored icons matched to categorization. Thus, the markers provide visual representation of the distribution and quality of food outlets within a 1km radius of each school site. 

The distance from the school sites to the nearest food outlets in each category was measured using Google Earth Pro and Google Maps. Google Maps provided an estimated time to reach these locations via different modes. For this study, the mode recorded was walking, the most common form of transport used by school students to visit a food outlet when moving to or from school. 

The frequency and distribution of food outlets across the two cases were reported. Relative healthy food access (RHFA), being healthy food outlets as a percentage of total food outlets, was calculated within each sector and overall. Variation in the distribution of outlets and categories within each sector in each country was assessed via one-sample Chi-Square test. The Mann–Whitney U test was used to determine whether there was a difference in the distribution and category of food outlets across the two sites [47]. 

The data underwent validation via quality checks. The food stores identified via on-the-ground observation were entered into Google Earth Pro. Where Google Mapping was at a level that was inclusive of retail outlets, a random sample of the Google Earth inputs was cross checked with Google Maps to confirm accuracy. Photographic evidence at the same randomly selected stores was used to cross-check categorisations. These checks were inclusive of 30 food outlets.

## 3. Results

**Figure 2:**
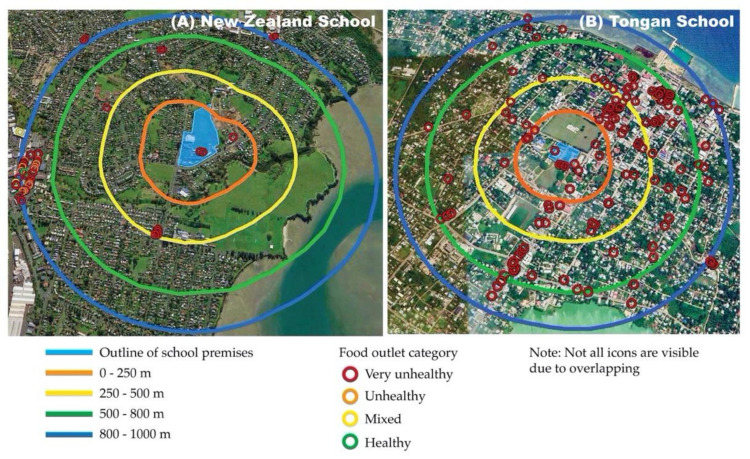
Food outlets within 1 km of (**A**) Setting A–the school in Auckland, NZ and (**B**), Setting B, the school in Nuku’alofa, Tonga; via Google Earth Pro 2017.

Visual assessment of the maps demonstrates that most outlets surrounding the schools are stocked with predominantly unhealthy foods. Differences in the quantity and distribution of food outlets surrounding each school are visible (Figure 2). 

### 3.1. Setting A: New Zealand

Fifty-two food outlets were observed within a 1 km radius of the Setting A school (Figure 2A). Distribution of outlets across sectors was unequal (𝜒^2^ (3) = 74.923, *p* < 0.001). Three outlets located within the 0–250 m zone included the ‘tuck-shop’ (a food outlet within school premises) and an onsite student-led café. On the 500 m border was a cluster of four outlets, located opposite a local primary school and kindergarten. Within the 500–800 m interval were five outlets comprising a single outlet (combined convenience/takeaway) and a cluster of four convenience-style stores. The distribution of categories of outlets across the sectors was unequal (𝜒^2^ (3) = 62.000, *p* < 0.001). All outlets in the 0 to 800 m zone were categorized as stocking predominantly very unhealthy foods (Table 4). The 800–1000 m zone contained three clusters of outlets. The largest cluster (35 outlets) sat within approximately 75% of the local shopping precinct. Of the 35 outlets in this precinct, 6% stocked predominantly healthy foods and 11% stocked a mix of healthy and unhealthy foods. The remaining outlets in the precinct stocked predominantly unhealthy or very unhealthy foods. 

The closest offsite food outlet beyond the school gate was a small convenience store located < 150 m, (3 min walk) from the boundary; classified as stocking predominantly very unhealthy food. The closest ‘healthy’ outlet was 1.1 km, approximately a 15 min walk from the school (Table 5).

### 3.2. Setting B: Tonga

One hundred and fifty food outlets were observed within a 1 km radius of the Setting B school (Figure 2B). Distribution across the sectors was unequal (𝜒^2^ (3) = 70.533, *p* < 0.001). Similarly, the distribution of categories of food outlets across the sectors was unequal (𝜒^2^ (3) = 282.280, *p* < 0.001). Of the 150 outlets, 98% were categorized ‘very unhealthy’, 0.7% ‘unhealthy’, and 1.3% ‘healthy’. The 12 outlets categorized as ‘very unhealthy’ within 250 m of the school included the school canteen. All 41 outlets within the 250–500 m radius were classified as ‘very unhealthy’. Within the 500–800 m, interval were 78 food outlets; 76 were categorized as ‘very unhealthy’, one ‘unhealthy’, and one ‘healthy’. The ‘healthy’ outlet was the primary fruit and vegetable market for the island, sited in the city centre. Within the 800 to 1000 m zone, 18 outlets were categorized as ‘very unhealthy’, and one as ‘healthy’. This was a daily street-side vegetable market (Table 4).

The closest offsite outlets were three food vendors that set up on the edge of the school premises, daily during school hours. All were categorized as ‘very unhealthy’. Adjacent to the school was a takeaway outlet. The closest ‘very healthy’ category outlet was almost 900 m from the school (Table 5). 

### 3.3. Setting Comparisons

A Mann–Whitney U test was run to determine if there were differences in the distribution of outlets between the two settings. Distribution of outlets across sectors was not similar, as assessed by visual inspection. Distribution by sector for Setting A, NZ (mean rank = 146.27) was statistically significantly higher than for Setting B, Tonga (mean rank 85.98), U = 6228.000, z = 6.777, *p* < 0.001.

Similarly, a Mann–Whitney U test determined that there were differences in the types of food outlets surrounding the two cases. Distribution of categorization of outlets across sectors was not similar, as assessed by visual inspection. Distribution of categories of food outlets for Setting A, NZ (mean rank = 121.43) was statistically significantly higher than for Setting B, Tonga (mean rank 94.59), U = 4936.500, z = 5.776, *p* < 0.001.

The overall RHFA measure was higher for Setting A, NZ, 3.85%, compared to Setting B, Tonga, 1.33%. However, in both cases RHFA was 0% in the school grounds and up to 500 m from the school. Both cases shared a RHFA of 5% in the 800–1000 m sector. This similarity is reflected in the 10–15 min walk to a food outlet categorized as stocking predominantly healthy foods for both cases. 

## 4. Discussion 

This study aimed to identify whether accessible, low-cost technologies could be used to map a point-in-time snapshot of the distribution and categorization of food outlets surrounding a school campus. Using Google Earth Pro supported by Google Maps, government maps (Tonga), and in person observations, maps indicating location and categorization of food outlets were constructed for both sites. 

No comparable mapping evidence is available for Tonga. Thus, we have assessed the validity of the method to enable credible data based on the NZ setting (School A). Day and Pearce [48] examined the distribution of fast-food and convenience outlets surrounding 63 secondary schools in five urban regions in NZ in 2008. They found variability in the number of fast-food and convenience outlets within the 800 m radial zone, (median= 4; max = 70; min = 0), with an average of 6.6 outlets per 1000 students [48]. School A (roll = 607 students) is situated in Auckland, one of the five urban regions represented in the Day and Pearce study [48]. Correcting for roll size, we might expect four fast-food or convenience outlets within the 800 m radial zone of School A. Twelve fast-food or convenience stores were identified within the 800 m zone, all categorized as stocking predominantly very unhealthy foods. Of the 12 outlets, two were within the school site; excluded for the purpose of comparison with the Day and Pearce study [48]. School A sits within the range of 0 to 70 outlets but is two and a half times higher than the average number of outlets per 1000 pupils in the 800 m zone reported by Day and Pearce [48]. School A is categorized by the NZ Ministry of Education as a Decile 1 school, indicating the highest possible level of socio-economic deprivation in the surrounding community [41]. Trends across all schools (primary to secondary inclusive) demonstrate higher numbers of fast-food and convenience outlets surrounding schools in low compared to high socioeconomic communities in NZ [38,48,49]. For example, within the 800 m radial buffer for data inclusive of all schools, Day and Pearce [48] identified that schools in the highest deprivation quintile had on average 2.5 times more fast-food and convenience outlets per 1000 students than schools in the lowest deprivation quintile [48]. We propose that the mapping data for School A sits within the expected range when compared to Day and Pearce [48] and aligns to the trend for more obesogenic retail food environments surrounding schools in low SES communities in NZ [38,48].

We sought to assess the potential for the methods to provide visualisable data to enable 13- to 17-year-old students to investigate the retail food environment surrounding their school. The method enabled the development of maps that provide visualisation of the distribution of outlets by radial zone and category. Clustering of outlets may limit the clarity of the data. However, viewed at a large size (as would be possible in a classroom setting) detail required to support exploration of patterns of distribution and category is possible. The visual nature of the data has the potential to be supportive of critical spatial thinking, a capability associated with examining and making sense of issues that are embedded in complex systems [50]. The visual data promotes pattern-seeking, a capability that is actively developed in learning areas across the New Zealand Curriculum [51]. Pattern-seeking links mathematical/statistical concepts with capabilities associated with scientific and health literacties which contribute to the potential for students to examine, make sense of and act on evidence [52]. The adequacy of the visual data to support inquiry-based learning cannot be assessed until classroom testing occurs. However, practicing teachers and educators on the study team assessed the maps as being valid to take to classroom testing in secondary schools in Tonga and NZ. 

A further outcome of the study is the evidence describing the retail food environment surrounding in each school, and the potential to make comparisons. Assessment based on absolute measures indicates that students from the school in Tonga were exposed to significantly more very unhealthy food outlets (n = 150) than those at the NZ School, *p* < 0.001. However, it is essential that context is considered when making comparisons. The school roll in School A, (607) is half that of School B, (1200). Correcting the data for school roll [48] we can identify very unhealthy and unhealthy food outlets at a rate of 75 per 1000 pupils in the map area for School A, compared to 123 per 1000 students for School B. However, School A is situated in a residential area, 1 km from the local shopping precinct while School B is situated within a predominantly commercial zone of a main thoroughfare, approximately 250 m from the edge of the city centre shopping precinct. Retail outlets and commercial premises stretch the full length of the street for 1 km north and south of the school. These differences highlight the importance of consideration of context. 

Analysis based on relative rather than absolute measures may be more important [20,21] and would be easily within the expected capability of secondary school students. Relative measures (Table 4) were calculated as ‘mixed + healthy/total’ and ‘healthy/total’ as it could be argued that healthy food is easily accessible in mixed-category outlets. Based on the mixed + healthy/total calculations, the measures suggest that the situation in Setting A, while still poor, is better than that for Setting B. However, the healthy/total measures indicate an equally poor retail food environment (RHFA, 0%) in the 0–500 m zone surrounding both schools. In the 800–1000 m zone the RHFA of 5% was shared by both schools. The overall healthy/total RHFA for Case B is one quarter of that for Case A. These relative measures point to the fact that students need to travel beyond the 500 m (Setting B) and 800 m (Setting A) zones to reach the few outlets that stock mixed or predominantly health foods. 

To our knowledge this is the first study to map a portion of the retail food environment within Nuku’alofa or any other area of Tonga. Globalization has increased the presence of imported foods in Tonga [53]. This is reflected in the retail food environment observed in this study and has been associated with increased reliance on processed foods and high intakes of fat, sugar and salt in many Pacific Island nations [54,55]. Factors influencing dietary choices are complex. The retail food environment is indicated as playing a role alongside individual and intrapersonal factors in adolescent food choices [56]. However, heterogeneous evidence is found regarding associations between the retail food environment, food choices and conditions such as obesity in adolescence [31,42]. The Setting B school is in an area that houses multiple primary and secondary schools. The high density of food outlets stocking predominantly unhealthy food options in the immediate vicinity of the school, combined with onsite food outlets and visiting lunchtime food vendors reflects a specific context. Given the influence of context (including culture) on food-related behaviours, we propose that there is potential value in expanding evidence of the retail food environment in and around schools in Tonga. Additionally, it would be valuable to investigate associations between food purchases from outlets within the school and passed on the journey to and from school, food choices and metabolic health. Additionally, the potential to engage adolescents in the conversation with politicians and the community leaders regarding the quality of the retail food environment surrounding schools should be examined. 

The use of Geographic Information systems (GIS) to promote learning has increased in secondary schools globally and in NZ [57,58]. GIS are identified as supporting problem- and inquiry-based learning, and promoting capabilities associated with critical spatial thinking [50,57,59]. Integrating GIS-based learning into exploration of the food environment has the potential to support engagement in inquiry based learning that promotes action-oriented outcomes. This reflects the importance of integration of the humanities and the social sciences in learning that promotes scientific and health literacies and develops capabilities associated with examining multiple perspectives when investigating complex socio-scientific issues [60]. While Google Earth Pro is not a true GIS, it is freely available and offers the tools associated with visualisation and mapping of geospatial data that was needed for this study [61]. 

Nutrition during adolescence is associated with future health of the individual and their potential children [62]. It is vital that autonomy and agency in relation to food choices by adolescents are recognized [63], and supported by appropriate educational opportunities [64]. Engaging adolescents in learning that examines and critiques evidence associated with food, nutrition and health can promote engagement in these complex issues, and in some cases contribute to sustained positive behavioural nudges associated with evidence-based decision-making [28,64]. This study provides a tool to enable teachers to integrate visual exploration of food environments into such a programme of learning. The inclusion of exploration of the retail the retail food environment alongside non-retail food environments (e.g., gardens), exploration of food-related behaviours within and beyond the school environment (access and consumption) and the impacts of nutrition in adolescence on lifelong health may offer potential to enhance the impact of learning of this nature. Collaboration to enable sharing of evidence between schools could offer the potential for adolescents to interrogate similarities and differences in retail food environments associated with geographic, socio-economic and cultural factors. We propose that the next phase of study should involve testing the hypothesis that, if supported with teacher professional development linked to health and education goals [27,29], the method used in this study may offer an opportunity to integrate health promotion into learning that promotes core curriculum objectives and can therefore be sustained within the school environment. 

A range of limitations are associated with this study. Testing the method in more than one site, particularly in NZ, would have strengthened the comparisons with previous mapping studies. The quality of the GIS is known to be a limiting factor in research of this nature [43]. Our aim was to enable mapping within freely available software, recognizing resource limitations. Google Earth provided the required features and was accessible in both contexts. It is possible that we missed some food outlets as data was collected from a single visit of the sites. This is more likely in Tonga where outlets may be mobile. The scoring system used to categorize the outlets was based on observations and may be subject to errors and biases held by the assessor. Finally, we acknowledge that we have only looked at one aspect of the retail food environment, omitting marketing, labelling policy, supply, and affordability. While these factors are vital to a full assessment, our focus in this study was intentionally limited to modelling of a mapping method that could be tested for use in schools. 

## 5. Conclusions

Using Google Earth Pro supported by Google Maps or government maps and in person observations, we demonstrated that maps showing distribution and categorization of the retail food environment surrounding a school campus can be constructed in geographically and technologically diverse settings using tools that are accessible to schools. We demonstrated that the data could be manipulated reasonably simply to report and potentially compare actual and relative retail food environments across two sites. The data emerging from the NZ setting was shown to reflect previously published trends associated with retail food environments surrounding NZ schools. The study has provided novel data relating to the retail food environment in Tonga, and identified alternative strategies that could be employed where features of the GIS-like system were not available. This study provides insight into tools that need to be tested in a classroom setting to assess their potential to support school-age students to engage in mapping and critiquing the retail food environment.

## Figures and Tables

**Table 1:** Rubric used to assess the quality of food available in each outlet.

Category	Food Type	Available in-Store (Tick)	Proportion of Food Stocked in the Store (%)	Healthfulness Rating
Health-promoting foods	Fresh fruit			
	Raw or salad vegetables			
	Cooked green vegetables			
	Starchy vegetables			
Risk promoting foods	Sweet stuff, candies, ice-cream			
	Muffins or cupcakes			
	Potato or corn crisps			
	Hot chips			
	Meat pies, sausage rolls			
Sugar-sweetened beverages	Chocolate milk/other flavored milk			
	Energy drinks			
	Regular fizzy or soft drinks			
	Diet fizzy or soft drinks			
	Juice, cordials and fruit drinks			

**Table 2:** Scoring system used for determining the healthfulness rating of a food outlet.

	Healthfulness Rating		
Proportion of Food Type Identified within the Store (%) (via Observation)	Health Promoting Foods	Risk Promoting Foods	Sugar-Sweetened Beverages
0–20	1	−1	−1
21–40	2	−2	−2
41–60	3	−3	−3
61–100	4	−4	−4

**Table 3:** Total healthfulness score categorization.

Total Score	Category	Map Icon
−4 to −7	Very unhealthy: Predominantly very unhealthy	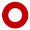
−1 to −3	Unhealthy: Predominantly unhealthy	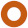
0 to 2	Mixed: Mix of healthy and unhealthy	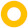
3 to 4	Healthy Predominantly health foods	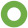

**Table 4:** Frequency of food outlets and relative healthy food access measure (RHFA); New Zealand (NZ), Tonga (TO).

	Food Outlet Categorization								Relative Healthy Food Access			
Radial Interval from School (m)	Very Unhealthy (n)		Unhealthy (n)		Mixed (n)		Healthy (n)		Mixed + Healthy/Total (%)		Healthy/Total (%)	
	NZ	TO	NZ	TO	NZ	TO	NZ	TO	NZ	TO	NZ	TO
0–250	3	12	0	0	0	0	0	0	0.00	0.00	0.00	0.00
250–500	4	41	0	0	0	0	0	0	0.00	0.00	0.00	0.00
500–800	5	76	0	1	0	0	0	1	0.00	1.28	0.00	1.28
800–1000	24	18	10	0	4	0	2	1	15.00	5.26	5.00	5.26
Total	36	147	10	1	4	0	2	2	11.5	1.33	3.85	1.33

**Table 5:** Distance from the school to the nearest food-outlet by category.

	Nearest Food Outlet in Each Health-Rating Category			
	Setting A: New Zealand		Setting B: Tonga	
Food Outlet Category	Distance (m)	Walk Time (min)	Distance (m)	Walk Time (min)
Very unhealthy	150	3	10	1
Unhealthy	965	12	944	12
Mixed	944	13		
Healthy	1100 *	15	883	10

* No outlet within 1000 m, distance to nearest ‘healthy’ food outlet.

## Data Availability

The data presented in this study are available within the article.
